# Signaling Pathways and Cellular Mechanisms Regulating Mossy Fiber Sprouting in the Development of Epilepsy

**DOI:** 10.3389/fneur.2018.00298

**Published:** 2018-05-03

**Authors:** Christin M. Godale, Steve C. Danzer

**Affiliations:** ^1^Department of Anesthesia, Cincinnati Children’s Hospital Medical Center, Cincinnati, OH, United States; ^2^Neuroscience Graduate Program, University of Cincinnati, Cincinnati, OH, United States; ^3^Department of Anesthesia, University of Cincinnati, Cincinnati, OH, United States; ^4^Department of Pediatrics, University of Cincinnati, Cincinnati, OH, United States

**Keywords:** mTOR, phosphatase and tensin homolog/PI3K/Akt, adult neurogenesis, dentate granule cell, epileptogenesis

## Abstract

The sprouting of hippocampal dentate granule cell axons, termed mossy fibers, into the dentate inner molecular layer is one of the most consistent findings in tissue from patients with mesial temporal lobe epilepsy. Decades of research in animal models have revealed that mossy fiber sprouting creates *de novo* recurrent excitatory connections in the hippocampus, fueling speculation that the pathology may drive temporal lobe epileptogenesis. Conducting definitive experiments to test this hypothesis, however, has been challenging due to the difficulty of dissociating this sprouting from the many other changes occurring during epileptogenesis. The field has been largely driven, therefore, by correlative data. Recently, the development of powerful transgenic mouse technologies and the discovery of novel drug targets has provided new tools to assess the role of mossy fiber sprouting in epilepsy. We can now selectively manipulate hippocampal granule cells in rodent epilepsy models, providing new insights into the granule cell subpopulations that participate in mossy fiber sprouting. The cellular pathways regulating this sprouting are also coming to light, providing new targets for pharmacological intervention. Surprisingly, many investigators have found that blocking mossy fiber sprouting has no effect on seizure occurrence, while seizure frequency can be reduced by treatments that have no effect on this sprouting. These results raise new questions about the role of mossy fiber sprouting in epilepsy. Here, we will review these findings with particular regard to the contributions of new granule cells to mossy fiber sprouting and the regulation of this sprouting by the mTOR signaling pathway.

Granule cells of the hippocampal dentate gyrus possess unique axonal projections known as mossy fibers. In healthy animals, mossy fibers produce numerous collaterals in the dentate hilus, which innervate mossy cells and hilar interneurons before projecting into stratum lucidum of the CA3 pyramidal cell layer, where they innervate interneurons (1) and the apical and basal dendrites of CA3 pyramidal cells (2). Recent work has also revealed that granule cells contact CA2 pyramidal cells, a population previously believed to be devoid of mossy fiber input (3–5). In laboratory animals modeling mesial temporal lobe epilepsy (mTLE), mossy fiber axon reorganization can occur in all of the hippocampal subfields normally targeted by granule cells (6–12). In addition to their normal projections, however, granule cell axons add an additional target to their repertoire: the inner molecular layer of the dentate gyrus (13–18). The dentate molecular layer contains the apical dendrites of the granule cells and is subdivided into inner, middle, and outer regions. Portions of the dendritic tree in the middle and outer molecular layers (oml) are innervated by afferents from entorhinal cortex. Axons from lateral entorhinal cortex favor the oml, while medial entorhinal cortex favors the middle (19, 20). The inner molecular layer contains the proximal-most portion of the granule cell dendritic trees, which are innervated by hilar interneurons, mossy cells, and commissural fibers (21, 22).

In the hippocampus, granule cell mossy fiber axons sprout, projecting new collaterals to the inner molecular layer, where they form excitatory synaptic connections with the proximal portions of the granule cell dendritic trees (23, 24). Mossy fiber sprouting is not indiscriminate, however, as the granule cell axons form a dense plexus of fibers in the inner molecular layer, but often avoid immediately adjacent regions like the granule cell body layer and the middle molecular layer. Co-occurring granule cell dispersion, in which the granule cell bodies spread into the molecular layer, does reduce this specificity. When robust dispersion is present, sprouted mossy fibers also target granule cell somas (25–27). The mechanisms regulating the specificity of mossy fiber sprouting have yet to be elucidated.

## Functional Significance of Mossy Fiber Sprouting in Epilepsy

Epilepsy is hypothesized to occur as a consequence of persistent changes in brain structure and function that create a propensity for spontaneous recurrent seizures (28). A key goal of epilepsy research, therefore, is to identify and understand the specific brain changes responsible for the disease. Mossy fiber sprouting “rewires” the epileptic hippocampus, creating recurrent excitatory circuits (24). The creation of this *de novo* pro-excitatory circuit, combined with its consistent appearance in humans and laboratory animals with mTLE, has led to an intense field of study aimed at determining whether mossy fiber sprouting plays a causal role in the disease.

Although mossy fiber sprouting is one of the most frequently observed changes in mTLE, the degree of sprouting varies considerably among laboratory animals and humans, ranging from extensive to undetectable (29). Schmeiser et al. (30), for example, observed no sprouting in 18% of patients with mTLE. The epilepsies include a complex array of dozens of identified disorders (31), so it is not a huge surprise that no single pathology is universally present, even within the sub-classification of mTLE. Furthermore, the absence of sprouting in some patients with mTLE clearly demonstrates that, at best, mossy fiber sprouting can only be relevant to a subset of patients with the condition, and is not *required* for the development of the disorder. Use of animal models has made it possible delve deeper into the relationship between sprouting and epilepsy, with investigators asking whether a positive correlation exists between the degree of sprouting and epilepsy severity. While many studies have revealed positive correlations, others have not (24). The circumstantial evidence linking mossy fiber sprouting to epilepsy, therefore, is ambiguous. One possible reason for inconsistent results is that mossy fiber sprouting is just one of the many pathologies that contribute to epilepsy. Hester and Danzer (32), for example, found that sprouting did positively correlate with seizure frequency in the rodent pilocarpine model of epilepsy. They also found, however, that positive correlations were even stronger when multiple pathologies were considered. This finding favors an “a la carte” model of epileptogenesis, in which epilepsy can be produced through different combinations of pathologies. If correct, sprouting may play a critical role in one patient, but be absent from another—even though both exhibit similar seizure phenotypes.

## Contributions of Newly Generated and Mature Granule Cells to Mossy Fiber Sprouting

Unlike the majority of neurons in the brain, dentate granule cells continue to proliferate throughout life in laboratory animals (33). Adult neurogenesis also appears to occur in humans (34, 35), although this conclusion is controversial (36). If neurogenesis does continue in adult humans, a population of neurons that differs considerably by age will result. These age differences are functionally significant, as young granule cells in rodents (4–6 weeks) exhibit morphological and physiological properties that are distinct from older cells (37–40). The impact of cell-age differences is also evident in epilepsy, where newborn granule cells are more likely to exhibit epilepsy-associated pathologies—such as misplacement to ectopic locations or formation of aberrant hilar-projecting basal dendrites—relative to adult granule cells (41–47). Notably, while discerning whether adult neurogenesis occurs in adult humans will take time and additional studies to resolve, mossy fiber sprouting and other granule cell abnormalities are clearly present in people with mTLE. These pathologies, therefore, will remain relevant regardless of their cellular origins.

The relationship between granule cell age and whether or not the cell contributes to mossy fiber sprouting has taken considerable effort to decipher. Initial studies clearly revealed that newborn granule cells contribute to mossy fiber sprouting (41), but numerous follow-up studies were required to establish that their contribution follows a complex temporal dynamic (44, 48, 49). Specifically, while some granule cell abnormalities, such as basal dendrites and ectopic localization, become evident before the newborn cells reach maturity (50–55), mossy fiber sprouting does not appear until the cells are functionally mature: about four weeks of age. The age range of granule cells that can contribute to mossy fiber sprouting is also broader than the range for other abnormalities. While ectopic cells originate almost exclusively from cells born after the epileptogenic insult, cells up to 7 weeks old at the time of the insult can contribute to mossy fiber sprouting (11). The broader age range of contributing cells, and the apparent requirement for cells to reach maturity before their axons begin to sprout, creates an age-gradient among the cells that underlie sprouting. The oldest cells (7 weeks) contribute first, with axons from these neurons appearing in the inner molecular layer within 2 weeks of the insult, while the axons of younger cells do not appear until 4 weeks after the insult.

The delayed development of mossy fiber sprouting by newborn granule cells is something of a surprise. This is because the delay does not reflect an inability of immature granule cells to produce an axon. Axogenesis begins within 4 days of granule cell birth (56), and axons reach stratum lucidum of CA3a—the distal-most region of CA3—by 12 days (57, 58). For a mossy fiber axon to reach CA3a, it must grow hundreds of microns further than would be needed to reach the inner molecular layer. Immature granule cells, therefore, are capable of extending axons significant distances, raising the question of why extension to the inner molecular layer is delayed till maturity. One plausible explanation is that immature cells are unresponsive to signals that induce mossy fiber sprouting among their more mature siblings. Examination of signaling molecules expressed at the transition from immature to mature stages could be a fruitful avenue for future studies.

## Impact of Ablating Newborn Cells on Mossy Fiber Sprouting

**Figure 1:**
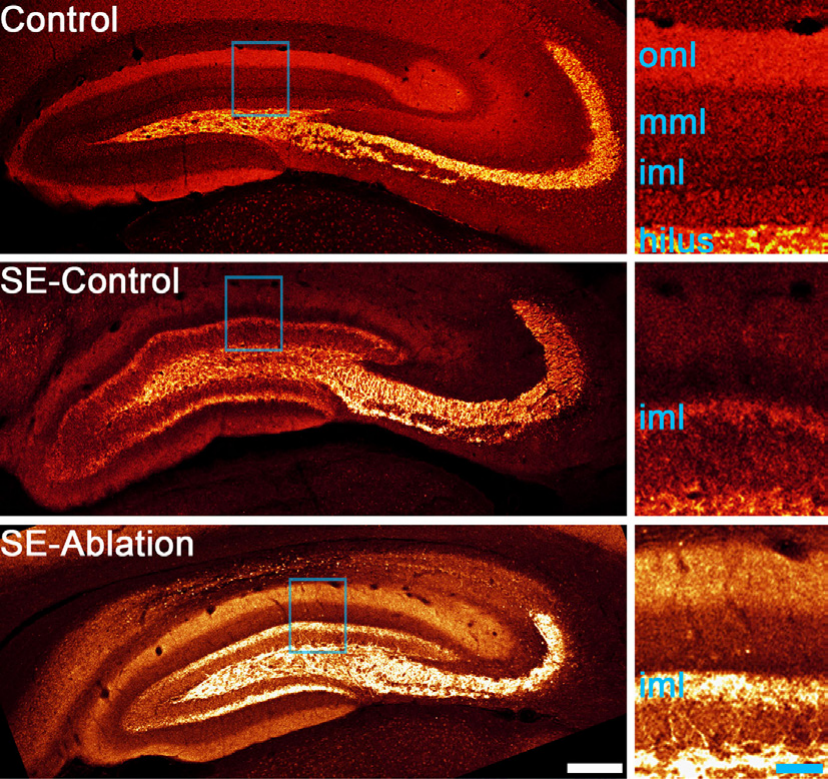
Confocal maximum projections showing mossy fiber axons labeled with zinc transporter-3 (red). A control animal and two epileptic animals are shown. Tissue from epileptic animals was collected about 5 months after pilocarpine-induced status epilepticus (SE). The epileptic animal shown in the middle panel received a control ablation treatment (SE-control), while newborn granule cells were ablated from the epileptic animal in the lower panel 1 month before tissue collection (SE-ablation). Regions highlighted in blue in each image are shown enlarged in the right panels. Both SE animals show mossy fiber sprouting in the inner molecular layer (iml), while the control animal did not. Newborn granule cell ablation reduced seizure frequency by about 50%, but had no effect on mossy fiber sprouting (61). Abbreviations: oml, outer molecular layer; mml, middle molecular layer. Scale bars = 200 µm (left) and 50 µm (right).

Advances in technology have made it possible to selectively manipulate newborn granule cells in the epileptic hippocampus to assess their role in epilepsy. In the first of these studies, Cho and colleagues (59) generated transgenic mice expressing the cell-killing gene thymidine kinase in granule cell progenitors. When they used these transgenic mice to block adult neurogenesis in the mouse pilocarpine model it significantly reduced spontaneous seizure frequency, but had no effect on the extent of mossy fiber sprouting. Similarly, Hosford and colleagues (60, 61) used a diphtheria-toxin receptor expression strategy to ablate newborn granule cells either days or months after pilocarpine epileptogenesis. Treatments reduced seizure occurrence by about 50%, but neither reduced mossy fiber sprouting (Figure 1). This dissociation raises questions about whether mossy fiber sprouting plays an important role in regulating seizure occurrence in epilepsy.

The absence of an effect of newborn cell ablation on the degree of mossy fiber sprouting was unexpected. The newborn granule cells eliminated have been shown to contribute to sprouting, so some reduction was predicted. Indeed, other measures of aberrant neurogenesis, like ectopic cell numbers, were reduced in all three studies (59–61). Explanations for the absence of an effect on sprouting include the possibility that the contribution of newborn cells is less than predicted, or that surviving cells compensate by increasing their level of sprouting. In the ablation experiments conducted to date, the interval between ablation and final analysis was several months, providing a window for compensatory cell growth to occur. Compensatory sprouting would also be consistent with the suggestion by Buckmaster (62) that homeostatic mechanisms act to maintain a target level of granule cell innervation in the brain, such that loss of recurrent input by ablation would induce surviving cells to replace that input. In either case, however, significant anti-epileptogenic effects can be achieved in the absence of reduced mossy fiber sprouting.

## Regulation of Mossy Fiber Sprouting by mTOR

The mechanistic (previously referred to as mammalian) target of rapamycin (mTOR) pathway has recently emerged as a promising target for anti-epileptogenesis therapies, and studies with mTOR antagonists provide some novel insights into the significance of mossy fiber sprouting. The mTOR pathway regulates neuronal survival, growth, and plasticity. In addition, the pathway becomes hyperactive following epileptogenic brain injury in animal models and in humans with epilepsy (63–65). In addition, many investigators have found that inhibition of the pathway with mTOR antagonists can reduce the incidence of spontaneous seizures in epileptic animals (66–71), although others have found no effect (72–75). The effects outlast the periods of drug exposure, indicative of disease-modifying, rather than acute anticonvulsant, properties (76). Evidence of disease-modifying effects raised the possibility that mTOR antagonism might mitigate pathological brain changes that mediate epileptogenesis, so naturally investigators assessed mossy fiber sprouting. Zeng and colleagues (66) observed reduced mossy fiber sprouting in the rodent kainic acid model of epilepsy when the animals were treated with the mTOR antagonist rapamycin; a finding replicated by numerous other groups using multiple epilepsy models (67–69, 71, 77–79). Direct infusion of rapamycin into the hippocampus also blocked sprouting locally around the infusion site (80). Notably, however, rapamycin can reduce mossy fiber sprouting without impacting seizures. Systemic treatment with rapamycin in the pilocarpine model of epilepsy blocked mossy fiber sprouting, but had no effect on seizure frequency (72, 74) or interictal spikes (81). Rapamycin was also ineffective at blocking paroxysmal discharges in the intrahippocampal kainic acid model of epilepsy, but did block mossy fiber sprouting (75). These findings are reminiscent of work begun decades ago, when it was found that treatment with the protein synthesis inhibitor cycloheximide could block mossy fiber sprouting, but not seizures in the rodent kainic acid and pilocarpine models (82–84). Although the efficacy of cycloheximide is controversial (85, 86), the finding that seizures can be dissociated from mossy fiber sprouting is not. Zhu et al. (87), for example, used the antineurogenic agent methylazoxymethanol acetate to reduce sprouting in the rodent pilocarpine model, but this had no effect on behavioral seizure frequency. Viewed in toto, therefore, rapamycin has consistently been shown to block mossy fiber sprouting, strongly implicating excess mTOR signaling in the process. Correlated reductions in mossy fiber sprouting and seizure frequency, however, occur in some cases but not in others, suggesting that sprouting may be an epiphenomenon, rather than a cause of mTLE.

**Figure 2:**
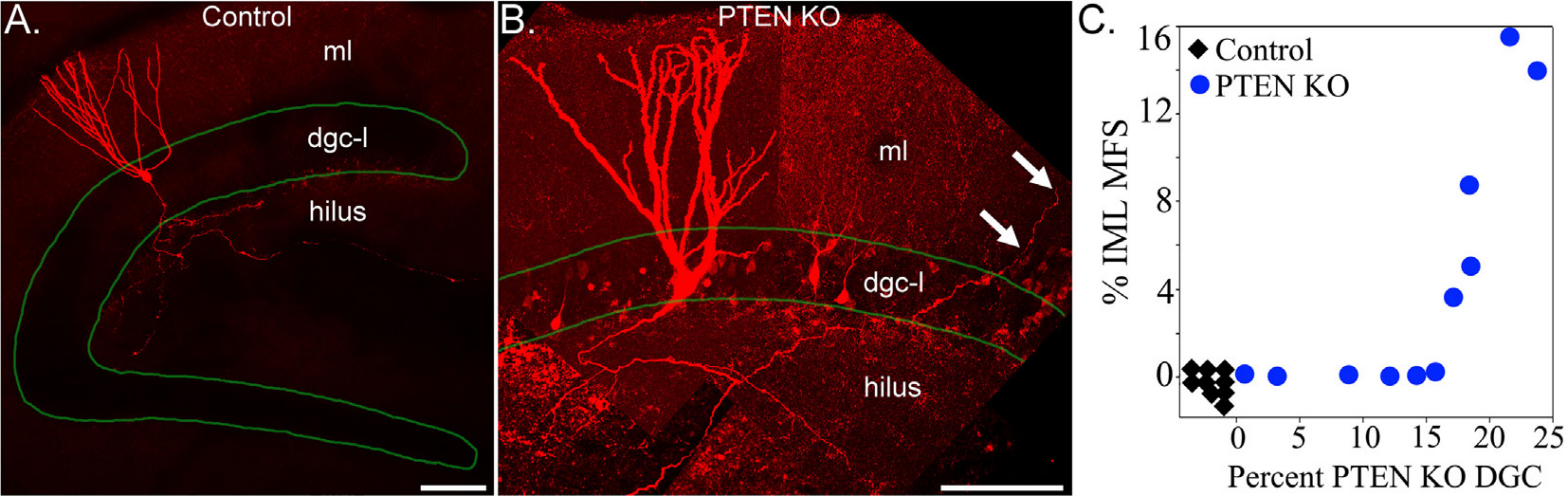
Confocal reconstructions of biocytin-filled hippocampal granule cells from a control **(A)** and a phosphatase and tensin homolog (PTEN) knockout **(B)** mouse. Labeled granule cells are shown in red, while the dentate granule cell body layer (dgc-l) borders are outlined in green. Note that the axons of the control cell are confined to the dentate hilus, while the PTEN knockout cell sends an axon collateral into the molecular layer (arrows). ml, dentate molecular layer. Scale bars = 100 µm. **(C)** Correlation between the degree of mossy fiber sprouting (mfs), assessed by ZnT3 immunoreactivity in the inner molecular layer (iml), and the percentage of PTEN KO granule cells. PTEN knockout cells and mossy fiber sprouting were absent from control animals (*n* = 9, black diamonds). Note the abrupt transition between animals with <15% PTEN knockout cells and no sprouting, and animals with deletion rates >15% and extensive sprouting. Figure reproduced in part from Pun et al. (89).

Analyses of transgenic animals with granule cell-specific hyperactivation of the mTOR pathway provide additional insights into the role of mTOR signaling in sprouting. Granule cell-specific hyperactivation of mTOR signaling has been achieved by deleting phosphatase and tensin homolog (PTEN) from granule cell progenitors and immature granule cells using tamoxifen-inducible Nestin-CreER^T2^, Gli1-CreER^T2^, and Pomc-Cre transgenic mice (88–90). PTEN acts as a negative regulator of the mTOR pathway, while the different promotors can be used to target PTEN deletion to postnatally generated granule cells. Deletion of PTEN from 2- to 4-week-old mice produces epilepsy in Nestin- and Gli1-Cre lines (88, 89). PTEN deletion leads to granule cell mossy fiber axon abnormalities, with evidence for increased axon collateralization in the hilus and stratum lucidum (91) and the development of recurrent excitation in acute hippocampal slices (92, 93). Epilepsy can develop in these animals, however, without robust mossy fiber sprouting (89). Sprouting does occur (Figure 2), but only in a subset of animals, demonstrating that sprouting is not essential for recurrent seizures in this model. Moreover, the degree of sprouting followed an “all or none” pattern, with some animals exhibiting extensive sprouting, and others essentially none (89). This result is interesting, because the percentage of granule cells lacking PTEN covered a broad range in this study: from 1 to 25%. If PTEN loss—and subsequent mTOR hyperactivation—leads to the direct, cell-intrinsic, induction of mossy fiber sprouting, one would predict a graded effect, with the degree of sprouting increasing gradually with the percentage of knockout cells. The absence of a graded increase implies that hyperactivation of the mTOR pathway is not sufficient to induce mossy fiber sprouting. Rather, the findings suggest some other factor is abruptly engaged once the animals reach deletion rates of about 15% (Figure 2). Whether this is the onset of spontaneous seizures or some other change remains to be determined.

## Therapeutic Implications

Mossy fiber sprouting is one of the most consistent pathological findings of mTLE, and the recurrent excitatory circuits created by sprouted mossy fiber axons have long held appeal as a mechanism of epileptogenesis. Recent work has provided new insights into the cellular and molecular basis of the phenomenon. First, it is now clear that both developmentally generated and adult-generated granule cells contribute to mossy fiber sprouting. The specific temporal dynamics of whether, and under what conditions, different age cell populations contribute is still being worked out, but it seems reasonable to conclude that therapies designed to reduce sprouting will need to target granule cells produced from development to adulthood. Second, mTOR pathway activation has emerged as an essential requirement of mossy fiber sprouting. With few exceptions (73), studies consistently demonstrate that blocking mTOR signaling prevents mossy fiber sprouting. mTOR hyperactivation by itself, however, does not appear to be sufficient to produce robust mossy fiber sprouting, as demonstrated by the absence of sprouting in some animals with enhanced mTOR signaling induced by PTEN loss. The mTOR pathway, therefore, appears necessary, but not sufficient, for mossy fiber sprouting. Finally, studies using a range of approaches have dissociated the degree of sprouting from seizure incidence. Epilepsy can occur in the absence of sprouting; sprouting can be reduced without impacting seizure incidence; and seizure incidence can be reduced without reducing sprouting. These negative findings, however, are not proof that mossy fiber sprouting is unimportant. Negative findings may reflect variable effects of sprouting in different epilepsy models, or even among patients. Sprouting may play a role in some cases and not others (32, 62). It is also possible that the relevance of mossy fiber sprouting is to epilepsy comorbidities rather than seizure incidence. People with mTLE and depression, for example, exhibit more sprouting that people with mTLE alone (94). The consistent, pronounced change in hippocampal wiring produced by mossy fiber sprouting almost certainly affects function, therefore, further study of the phenomenon is critical.

## Author Contributions

SD and CG: wrote manuscript, conducted literature searches, and prepared figures.

## Conflict of Interest Statement

The authors declare that the research was conducted in the absence of any commercial or financial relationships that could be construed as a potential conflict of interest.
